# The first mitochondrial genome of *Haemagogus equinus* from Jamaica

**DOI:** 10.12688/f1000research.159115.2

**Published:** 2025-07-02

**Authors:** Simone L. Sandiford, Simmoy A. A. Noble, Samra A. Pierre, Douglas E. Norris, Renee Ali

**Affiliations:** 1 Department of Basic Medical Sciences, Pharmacology and Pharmacy Section, Faculty of Medical Sciences, The University of the West Indies Mona, Kingston, Jamaica; 2 Mosquito Control and Research Unit, The University of the West Indies, Mona, Kingston, Jamaica; 3 Department of Microbiology, Faculty of Medical Sciences, The University of the West Indies Mona, Kingston, Jamaica; 4 Department of Life Sciences, Faculty of Science & Technology, The University of the West Indies Mona, Kingston, Jamaica; 5 The W. Harry Feinstone Department of Molecular Microbiology and Immunology, Johns Hopkins Malaria Research Institute, Johns Hopkins Bloomberg School of Public Health, Baltimore, Maryland, USA

**Keywords:** Haemagogus equinus, mitogenome, Jamaica, arboviruses, genome skimming

## Abstract

In the Americas, the expansion in incidence of arboviral infections including Mayaro virus (MAYV) has drawn attention to the resurgence of viruses associated with understudied arthropods. Mosquitoes belonging to the genus *Haemagogus* are generally geographically restricted to the forests of Central and South America and the Caribbean and are the known sylvan vectors for yellow fever virus and emerging MAYV. With an established population in Jamaica, *Haemagogus equinus* has been reported to be well-adapted to oviposition in artificial containers close to human populations. Its role in arboviral transmission however is not fully understood. Given the dearth of genetic information and the difficulty in morphologically identifying cryptic features in species belonging to this genus, we report the first mitochondrial genome of *Hg. equinus.* Using a genome skimming approach, two *Hg. equinus* mosquito specimens were sequenced using the Illumina Novaseq 6000 platform. A representative mitogenome of 16,471 bp, 80.7% AT and 37 genes was assembled using NOVOplasty. Phylogenetic analysis placed *Hg. equinus* in the Albomaculatus section of the *Haemagogus* subgenus supporting previously described taxonomic studies.

## Introduction

Mosquitoes of the genus *Haemagogus (Hg.)* Williston, 1896 are endemic to tropical rainforests, open deciduous forests and mangroves in Latin America and the Caribbean. ^1^ The genus is divided into two subgenera ( *Haemagogus* and *Conopostegus*), with the *Haemagogus* subgenus split into three sections (Albomaculatus, Splendens, Tropicalis). ^1^ Being the primary sylvatic vectors for yellow fever virus (YFV) ^1^ and the emerging arboviral threat Mayaro virus (MAYV), ^2^ species in this genus of mosquitoes are of significant medical importance in the region. Despite this, many species remain understudied and their role in disease transmission has not been clearly defined. In Jamaica, *Haemagogus equinus* Theobald, 1903 is presently the only known species on the island. Established populations of *Hg. equinus* have also been identified in southern Texas in the United States of America, Mexico, Belize, Guatemala, El Salvador, Honduras, Nicaragua, Costa Rica, Panama, Colombia, Guyana, Venezuela and Trinidad and Tobago. ^1^

While the vectorial capacity of *Hg. equinus* has not been fully elucidated, laboratory and transovarial transmission of YFV ^3– 6^ along with natural infections among wild mosquitoes have been reported. ^7, 8^ In spite of this, its importance in arboviral transmission in some localities remains uncertain. ^9^ Although *Hg. equinus* primarily oviposits and larvae develop in tree holes and bamboo internodes, ^1^ the species is very adaptable and can also utilize rock holes, ^10^ domestic containers and used tires. ^11, 12^ With multiple reports of MAYV in neighbouring Haiti, ^13– 15^ there is need for a greater understanding of the role of this sylvatic vector in arbovirus transmission. To facilitate this, accurate identification of all life stages of field collected specimens is crucial. However, impediments such as a lack of taxonomic expertise coupled with poor quality samples and a paucity of reference molecular data continue to hamper these efforts in Jamaica.

Few studies have employed genomic sequencing to investigate the molecular taxonomy and phylogenetic relationships of *Hg. equinus* mosquitoes. Currently, only partial sequences of *cytochrome oxidase I* and other mitochondrial or nuclear genes are available from NCBI’s GenBank or BOLD systems databases. ^16– 21^ The complete mitochondrial genome (mitogenome) is increasingly being used for evolutionary and phylogeny studies due to its maternal inheritance, simple genomic organization, relative abundance in tissues and absence of recombination. ^22^ In most metazoans the mitogenome is highly conserved and comprises of 13 protein coding genes, 22 transfer RNAs and two ribosomal RNAs in addition to a large single non-coding region important for replication and transcription. ^23^ At present, mitogenomes of only five species from the genus *Haemagogus* are available. ^24, 25^ Considering the significance of mitogenomes when conducting phylogenetic and taxonomic studies, we describe for the first time the characterization of the mitogenome of *Hg. equinus* using a genome skimming approach and the phylogenetic comparison with other *Haemagogus* taxa.

## Methods

The *Hg. equinus* samples (n = 2) sequenced were collected in Mona, Jamaica (18.0061364°N, -76.7515125°W) in May and December 2023 in an area characterized by the predominant growth of banana plants and adjacent to a forested area. Briefly, BG sentinel traps (BioGents, Regensburg, Germany) baited with two pounds of dry ice (without lure) were placed overnight from 1400 to 1000 hr and collected mosquito specimens were sorted and morphologically identified using taxonomic keys. ^26^ Single specimens were stored in tubes containing silica and shipped to the Johns Hopkins Bloomberg School of Public Health for molecular analysis as described in detail in. ^27^ Briefly, each mosquito specimen was homogenized in PK buffer (Applied Biosystems, Waltham, USA) containing proteinase K (Applied Biosystems, Waltham, USA) and incubated at 56°C. DNA was then extracted using the Qiagen DNeasy Blood and Tissue Kit (Hilden, Germany) and quantified using the Qubit dsDNA assay kit (ThermoFisher, Waltham, USA) prior to library construction and Illumina sequencing which was conducted at SeqCenter (Pittsburgh, USA).

**Figure 1.**
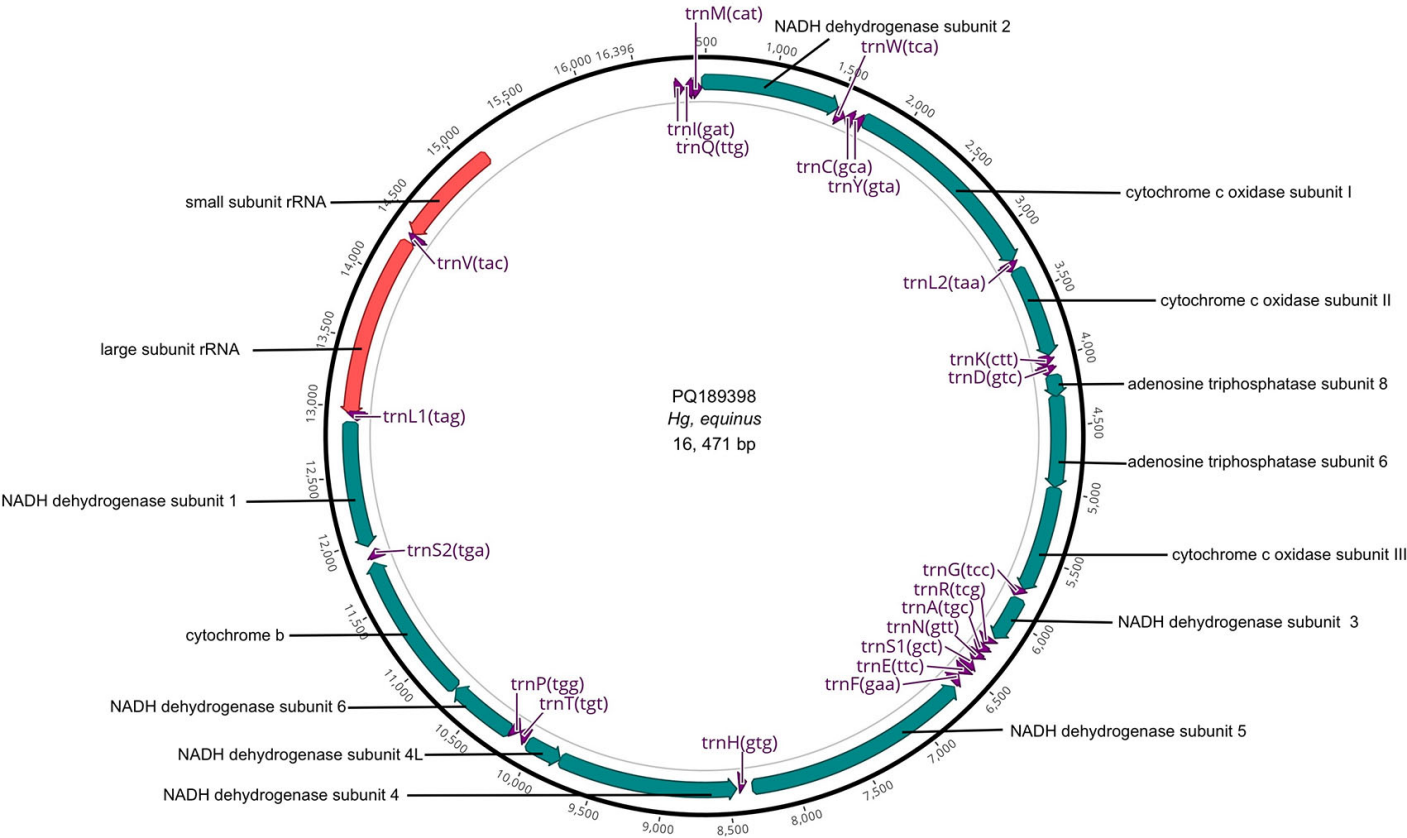
Mitogenome map of *Hg. equinus* with annotated genes.

The mitogenome was assembled as described for African anophelines using NOVOPlasty (RRID:SCR_017335) version 4.3.1 ^27, 28^ and automatic annotations performed with MITOS on the galaxy platform under the invertebrate genetic code. ^29, 30^ Geneious Prime (RRID:SCR_010519) version 2023.2.1 (Biomatters, Auckland, Australia) was utilized for manual adjustments of start and stop codons to match reference *Haemagogus* mitogenomes in the GenBank repository. Figure 1 illustrates a representative map of the mitochondrial sequences and annotations submitted to GenBank.

Nine mitogenomes (two sequenced from this study and seven available from GenBank) were used for phylogenetic analysis. The 13 protein coding genes of these mitogenomes were extracted with Geneious Prime and aligned by MAFFT version 7.490 into a single matrix. jModelTest version 2.1.10 identified the best fit substitution model and phylogenetic analyses were performed using maximum likelihood in MEGA (RRID_SCR_023017) version 11 with 1000 bootstrap replicates.

## Results

**Figure 2.**
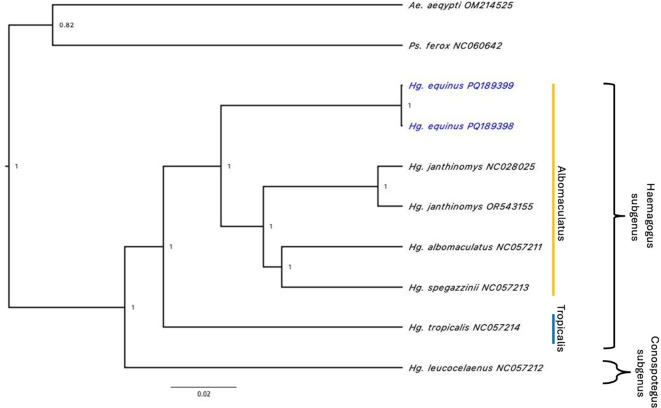
Maximum likelihood tree using the General Time Reversible (GTR + G + 1) substitution model based on the 13 concatanated protein coding genes of *Hg. equinus*, six *Haemagogus* species and *Psorophora ferox* von Humboldt, 1819 and *Aedes aegypti* Linnaeus, 1762 as outgroups. *Haemagogus*, *Psorophora* and *Aedes* are all members of the tribe Aedini.

Sequencing of the two *Hg. equinus* specimens resulted in a mean of 26,728,491 million reads and of these approximately 69,261 reads were utilized for assembling each mitogenome. The two specimens shared 98.84% similarity. Two ribosomal RNAs, 22 transfer RNAs and 13 protein coding genes were detected in the two *Hg. equinus* mitochondrial genomes (GenBank accession numbers PQ_189398, PQ_189399). The *cytochrome c oxidase I* (COI) segment covering 1691-3221 bp was 99.59 % comparable to a COI sequence for *Haemagogus spegazzinii* Brèthes, 1912 (YP_010155459) retrieved from GenBank. Similar to the six *Haemagogus* mitochondrial genomes available in the GenBank database, the representative mitogenome from our sequencing efforts (PQ_189398) has an A+T proportion of 80.7% and length of 16,471 bp. The Maximum Likelihood phylogenetic tree places it in the Albomaculatus section, separated from other *Haemagogus* mitogenomes ( Figure 2).

These findings provide the basis for the development of more accurate molecular tools which can be used for identification of *Hg. equinus* mosquitoes. Furthermore, by populating molecular repositories with genomic data from Jamaican mosquitoes, their phylogenetic and evolutionary status will be better understood.

## Data availability

GenBank: *Haemagogus equinus* mitochondrion, complete genome. Accession numbers PQ189398, PQ189399; https://identifiers.org/ncbi/insdc:PQ189398, https://identifiers.org/ncbi/insdc:PQ189399. ^31^

Bio Project. Complete mitochondrial genome of *Haemagogus equinus*, Accession number PRJNA1172362; https://www.ncbi.nlm.nih.gov/bioproject/PRJNA1172362. ^32^

SRA. Illumina seq of *Haemagogus equinus.* Accession numbers SRR31002245, SRR31002246; https://www.ncbi.nlm.nih.gov/sra/?term=PRJNA1172362. ^33^

Biosample: *Haemagogus equinus* isolates HGJ1, HGJ3. SAMN44269491, SAMN44269492; https://trace.ncbi.nlm.nih.gov/Traces/study/?acc=PRJNA1172362. ^34^

### Ethics and consent

Ethical approval and consent were not required.

## References

[1] Arnell JH : Mosquito studies (Diptera, Culicidae). XXXII. A revision of the genus Haemagogus. *Contributions of the American Entomological Institute.* 1973;10:ii + 1–ii + 174.

[2] Pan American Health Organization: Epidemiological Alert: Mayaro Fever.(accessed on 15 August). Reference Source

[3] Waddell MB Taylor RM : Studies on Cyclic Passage of Yellow Fever Virus in South American Mammals and Mosquitoes: Marmosets (Callithrix aurita) and Cebus Monkeys (Cebus versutus) in Combination with Aedes aegypti and Haemagogus equinus. *Am. J. Trop. Med.* 1945;s1-25:225–230. 10.4269/ajtmh.1945.s1-25.225

[4] Waddell MB Taylor RM : Studies on the Cyclic Passage of Yellow Fever Virus in South American Mammals and Mosquitoes: III. Further Observations on Haemagogus equinus as a Vector of the Virus. *Am. J. Trop. Med.* 1947;s1-27:471–476. 10.4269/ajtmh.1947.s1-27.471

[5] Waddell MB : Comparative Efficacy of Certain South American Aëdes and Haemagogus Mosquitoes as Laboratory Vectors of Yellow Fever. *Am. J. Trop. Med.* 1949;29:567–575. 10.4269/ajtmh.1949.s1-29.567 18153054

[6] Dutary BE Leduc JW : Transovarial transmission of yellow fever virus by a sylvatic vector, Haemagogus equinus. *Trans. R. Soc. Trop. Med. Hyg.* 1981;75:128. 10.1016/0035-9203(81)90036-5 6115487

[7] De Rodaniche E Galindo P : Isolation of yellow fever virus from Haemagogus mesodentatus, H. equinus and Sabethes chloropterus captured in Guatemala in 1956. *Am. J. Trop. Med. Hyg.* 1957;6:232–237. 10.4269/ajtmh.1957.6.232 13424899

[8] De Rodaniche E Galindo P Johnson CM : Isolation of yellow fever virus from Haemagogus lucifer, H. equinus, H. spegazzinii falco, Sabethes chloropterus and Anopheles neivai captured in Panama in the fall of 1956. *Am. J. Trop. Med. Hyg.* 1957;6:681–685. 10.4269/ajtmh.1957.6.681 13458674

[9] Chadee DD Hingwan JO Persad RC : Seasonal abundance, biting cycle, parity and vector potential of the mosquito Haemagogus equinus in Trinidad. *Med. Vet. Entomol.* 1993;7:141–146. 10.1111/j.1365-2915.1993.tb00667.x 8097636

[10] Chadee DD : Rock hole breeding Haemagogus mosquitoes on Monos Island, Trinidad, West Indies. *Mosq. News.* 1983;43:236–237.

[11] Chadee DD Maitre A l Connell NK : The collection of Haemagogus equinus Theobald breeding in household containers in Tobago WI. *Mosq. News.* 1981;41:568–569.

[12] Parra C Liria J : Haemagogus equinus Theobald 1903 (Diptera: Culicidae) en el Campus de la Universidad de Carabobo. Valencia. Venezuela. *Salus.* 2010;14:41–43.

[13] Blohm G Elbadry MA Mavian C : Mayaro as a Caribbean traveler: Evidence for multiple introductions and transmission of the virus into Haiti. *Int. J. Infect. Dis.* 2019;87:151–153. 10.1016/j.ijid.2019.07.031 31382049 PMC9527705

[14] Lednicky J De Rochars VMB Elbadry M : Mayaro virus in child with acute febrile illness, Haiti, 2015. *Emerg. Infect. Dis.* 2016;22:2000–2002. 10.3201/eid2211.161015 27767924 PMC5088037

[15] White SK Mavian C Elbadry MA : Detection and phylogenetic characterization of arbovirus dual-infections among persons during a chikungunya fever outbreak, Haiti 2014. *PLoS Negl. Trop. Dis.* 2018;12:e0006505. 10.1371/journal.pntd.0006505 29851952 PMC5997359

[16] Chan-Chable RJ Martínez-Arce A Mis-Avila PC : DNA barcodes and evidence of cryptic diversity of anthropophagous mosquitoes in Quintana Roo, Mexico. *Ecol. Evol.* 2019;9:4692–4705. 10.1002/ece3.5073 31031936 PMC6476762

[17] Gibson CM Kao RH Blevins KK : Integrative Taxonomy for Continental-Scale Terrestrial Insect Observations. *PLoS One.* 2012;7:e37528. 10.1371/journal.pone.0037528 22666362 PMC3362597

[18] Adeniran AA Hernández-Triana LM Ortega-Morales AI : Identification of mosquitoes (Diptera: Culicidae) from Mexico State, Mexico using morphology and COI DNA barcoding. *Acta Trop.* 2021;213:105730. 10.1016/j.actatropica.2020.105730 33096064

[19] Viveros-Santos V Hernández-Triana LM Ibáñez-Bernal S : Integrated Approaches for the Identification of Mosquitoes (Diptera: Culicidae) from the Volcanoes of Central America Physiographic Subprovince of the State of Chiapas, Mexico. *Vector borne and zoonotic diseases (Larchmont, N.Y.).* 2022;22:120–137. 10.1089/vbz.2021.0034 35175140

[20] Hernández-Triana LM Garza-Hernández JA Ortega Morales AI : An Integrated Molecular Approach to Untangling Host–Vector–Pathogen Interactions in Mosquitoes (Diptera: Culicidae) From Sylvan Communities in Mexico. *Front. Vet. Sci.* 2021;7. 10.3389/fvets.2020.564791 33778029 PMC7988227

[21] Reidenbach KR Cook S Bertone MA : Phylogenetic analysis and temporal diversification of mosquitoes (Diptera: Culicidae) based on nuclear genes and morphology. *BMC Evol. Biol.* 2009;9:298. 10.1186/1471-2148-9-298 20028549 PMC2805638

[22] Cameron SL : Insect mitochondrial genomics: implications for evolution and phylogeny. *Annu. Rev. Entomol.* 2014;59:95–117. 10.1146/annurev-ento-011613-162007 24160435

[23] Boore JL : Animal mitochondrial genomes. *Nucleic Acids Res.* 1999;27:1767–1780. 10.1093/nar/27.8.1767 10101183 PMC148383

[24] Silva FS da Cruz ACR Almeida Medeiros DB de : Mitochondrial genome sequencing and phylogeny of Haemagogus albomaculatus, Haemagogus leucocelaenus, Haemagogus spegazzinii, and Haemagogus tropicalis (Diptera: Culicidae). *Sci. Rep.* 2020;10:16948. 10.1038/s41598-020-73790-x 33046768 PMC7550346

[25] Lemos PS Monteiro HAO Castro FC : Characterization of mitochondrial genome of Haemagogus janthinomys (Diptera: Culicidae). *Mitochondrial DNA Part A.* 2017;28:50–51. 10.3109/19401736.2015.1110793 26709451

[26] Belkin JN Heinemann SJ Page WA : The Culicidae of Jamaica (Mosquito Studies. XXI). *Contrib. Am. Entomol. Inst.* 1970;6:1–458.

[27] Ali R Gebhardt M Lupiya J : The first complete mitochondrional genome of Anopheles gibbinsi using a skimming sequencing approach. [version 1; peer review: 3 approved]. *F1000Res.* 2024;13:553. 10.12688/f1000research.148473.1 39036652 PMC11258543

[28] Dierckxsens N Mardulyn P Smits G : NOVOPlasty: de novo assembly of organelle genomes from whole genome data. *Nucleic Acids Res.* 2017;45:e18. 10.1093/nar/gkw955 28204566 PMC5389512

[29] Bernt M Donath A Jühling F : MITOS: improved de novo metazoan mitochondrial genome annotation. *Mol. Phylogenet. Evol.* 2013;69:313–319. 10.1016/j.ympev.2012.08.023 22982435

[30] Afgan E Baker D Batut B : The Galaxy platform for accessible, reproducible and collaborative biomedical analyses: 2018 update. *Nucleic Acids Res.* 2018;46:W537–W544. 10.1093/nar/gky379 29790989 PMC6030816

[31] Noble SAA Pierre SA Sandiford SL : Haemagogus equinus mitochondrion, complete genome.[Dataset]. *GenBank.* 2024.

[32] Johns Hopkins Bloomberg School of Public Health: Complete mitochondrial genome of Haemagogus equinus.[Dataset]. *Bioproject.* 2024.

[33] Johns Hopkins Bloomberg School of Public Health: llumina seq of Haemagogus equinus.[Dataset]. *SRA.* 2024.

[34] Johns Hopkins Bloomberg School of Public Health: Invertebrate sample of Haemagogus equinus.[Dataset]. *Biosample.* 2024.

