# Supplementary material for: The first mitochondrial genome of Haemagogus equinus from Jamaica
Source: F1000Res. 2025 Jul 2;13:1504. Originally published 2024 Dec 9. [Version 2] doi: 10.12688/f1000research.159115.2 (PMC12586976; doi:10.12688/f1000research.159115.2)
Supplement: Peer review history

# Reviewer report: Reviewer response for version 2

**DOI:** 10.5256/f1000research.184140.r421208

**Published:** 2025-11-04

**Authors:** Alexey Makunin

**Affiliations:** 1 Wellcome Sanger Institute, Hinxton, England, UK

The manuscript by Sandiford, et al., describes two mitochondrial genomes for arboviral vector mosquito *Haemagogus equinus*. The dataset is succinctly described and characterised to ensure that the resulting mitochondrial genomes become a valuable resource for future studies. I only have several minor comments:

 - Illumina read length and layout (single or paired end) are not specified

 - "manual adjustments of start and stop codons to match reference _ *Haemagogus*_ mitogenomes in the GenBank repository" - if the sequencing effort did not allow to accurately reconstruct start and stop codon, the accuracy of the remaining sequence and subsequent phylogenetic analysis can be compromised

 - Selected optimal substitution model for phylogenetic analysis is not specified. Also, it seems that the entire matrix was treated as a single partition, without considering separate models for different codon positions - is it correct?

 - COI analysis looks a bit confusing - the coordinates are given for the entire gene, not the ~658bp fragment used for barcoding. "99.59 % comparable to a COI sequence for *Haemagogus spegazzinii *(YP_010155459)" seemingly indicates protein identity for 510aa segment in YP_010155459, which does not seem relevant for species identification. While this might be out of scope for this particular study (given the complete MT PCG tree looks convincing and *Hg. equinus* does not seem to be closely related to any other species with complete MT available), I would suggest building a tree based on nucleotide sequence of COI barcode for all Hg. records in BOLD with extracts from two new mitogenomes added - this would complement and verify the PCG tree results with more observations, but smaller resolution.

Are the datasets clearly presented in a usable and accessible format, and the assembly and annotation available in an appropriate subject-specific repository?

Yes

Are sufficient details of the sequencing and extraction, software used, and materials provided to allow replication by others?

Yes

Are the rationale for sequencing the genome and the species significance clearly described?

Yes

Are the protocols appropriate and is the work technically sound?

Yes

Reviewer Expertise:

Comparative genomics, chromosome evolution

I confirm that I have read this submission and believe that I have an appropriate level of expertise to confirm that it is of an acceptable scientific standard.

# Reviewer report: Reviewer response for version 1

**DOI:** 10.5256/f1000research.174799.r349839

**Published:** 2025-01-29

**Authors:** Craig Wilding

**Affiliations:** 1 School of Biology and Environmental Science, University College Dublin, Dublin, Leinster, Ireland

Sandiford et al sequence full mitochondrial genomes from genome skimming data from two *Haemagogus equinus* specimens collected from Jamaica. They assemble these into a full mtDNA genome containing the expected gene content. Their introduction is well-written and provides much background information and adequate justification for this study. However, I would argue that the statement in the introduction that “Few studies have employed genomic sequencing to investigate the molecular taxonomy and phylogenetic relationships of *Hg. equinus* mosquitoes” suggests that this paper is going to provide a genome. Whilst it could be argued that the mtDNA is a genome it is somewhat misleading!

 In the Methods there is no information on the sequencing undertaken. Whilst the authors say “molecular analysis as described in [27]” this is insufficient – at the very least there should be a brief description of what was done.

 The statement “Nine mitogenomes (two sequenced from this study and seven available from GenBank) were used for analysis” should read “Nine mitogenomes (two sequenced from this study and seven available from GenBank) were used for phylogenetic analysis”

 In the Results section, Figure 1 has a very poor resolution. This needs altering. Figure 2 has the albomaculatus and tropical groups highlighted, but which group does *Hg. leucocelaenus* belong? Is this Splendens? Or if not, are there no mitochondrial genomes from this group? I am not particularly clear on the point of Figure 2.

 It would make more sense to produce a tree on a wider-scale (including e.g. representative *Aedes* and *Sabethes* or, have a tree to show the reader the position of *Hg. equinus *within the wider Aedini and then have this zoomed in view of *Haemagogus*, but for those readers unfamiliar with this genus the Figure does not really say anything of use and the text merely says “The Maximum Likelihood phylogenetic tree places it in the Albomaculatus section”. Was this not known before these mitochondrial sequences were generated?

 The authors make no comment on whether the gene order seen in this species is as expected given those of closely related species. They should do so.

 This study sequenced two specimens with accession numbers PQ_189398 and PQ_189399. How different were they? Some indication of overall genetic distance would be useful.

Are the datasets clearly presented in a usable and accessible format, and the assembly and annotation available in an appropriate subject-specific repository?

Yes

Are sufficient details of the sequencing and extraction, software used, and materials provided to allow replication by others?

No

Are the rationale for sequencing the genome and the species significance clearly described?

Yes

Are the protocols appropriate and is the work technically sound?

Yes

Reviewer Expertise:

Evolutionary genetics

I confirm that I have read this submission and believe that I have an appropriate level of expertise to confirm that it is of an acceptable scientific standard.

# Response

**Published:** 2025-06-24

**Authors:** Simone Sandiford

**Affiliations:** Basic Medical Sciences, The University of the West Indies at Mona, Mona, Saint Andrew, Jamaica

Thank you for your insightful comments regarding our manuscript.  As suggested, we have replaced Figure 1 with a high quality version, enhanced Figure 2, added further details regarding the molecular analysis to the methods section and stated the similarity between the 2 sequenced specimens in the results.

# Reviewer report: Reviewer response for version 1

**DOI:** 10.5256/f1000research.174799.r349846

**Published:** 2025-01-08

**Authors:** Din Syafruddin, Puji Budi Setia Asih

**Affiliations:** 1 National Research and Innovation Agency (BRIN), Makassa, Indonesia; 2 Eijkman Research Center for Molecular Biology, National Research and Innovation Agency, Indonesia, Indonesia

**General comment:**

 The manuscript reports the complete mitochondrial genome sequence of *Haemagogus equinus* from Jamaica.  The genome is circular and includes 13 protein-coding genes, 2 ribosomal RNA genes and  22 tRNA genes. As the member of tribe Aedini and vector of Yellow fever, it might be informative to include mitochondrial genome of *Aedes aegypti *in the phylogenetic tree.

**Specific comments:**

1. The map should clearly indicate the position of the 13 protein coding genes, rRNA genes and 22 tRNA genes in the light and heavy strands of mtDNA (Fig. 1).       
2. The authors should include “discussion” section in the manuscript to describe the characteristic of the genes encode by mitochondrial gnome of the Haemogogus equinus in comparison to cosmopolitan species of the tribe Aedini, *Aedes aegypti*.  

Are the datasets clearly presented in a usable and accessible format, and the assembly and annotation available in an appropriate subject-specific repository?

Yes

Are sufficient details of the sequencing and extraction, software used, and materials provided to allow replication by others?

Yes

Are the rationale for sequencing the genome and the species significance clearly described?

Yes

Are the protocols appropriate and is the work technically sound?

Yes

Reviewer Expertise:

NA

We confirm that we have read this submission and believe that we have an appropriate level of expertise to confirm that it is of an acceptable scientific standard, however we have significant reservations, as outlined above.

# Response

**Published:** 2025-01-23

**Authors:** Simone Sandiford

**Affiliations:** Basic Medical Sciences, The University of the West Indies at Mona, Mona, Saint Andrew, Jamaica

Thank you to the reviewers for the feedback provided.

 Our article was submitted as a genome note.  Based on the F1000Research guidelines genome notes only require an introduction and a detailed protocol in the methods section which discusses the sequencing of the genome.  We have fulfilled both requirements.  A results section is optional, and we have chosen to provide this also.  A discussion section is not needed hence it was not included. 

 As it relates to the tree, *Psorophora ferox* was used as an outgroup as it is also a member of tribe Aedini and like *Haemagogus equinus*, is a forest mosquito.

 Figure 1 was constructed similar to other illustrated figures in genome notes which have previously been published and unanimously approved by peer reviewers in F1000Research as indicated below.

 https://f1000research.com/articles/12-330

https://f1000research.com/articles/13-553
